# Dr. Baillie, in Answer to Dr. Hull

**Published:** 1802-02

**Authors:** M. Baillie

**Affiliations:** Great Windmill Street


### *To the Editors of the Medical and Phyfical Journal.*

GENTLEMEN,

I HAVE read in your ufeful Journal the Obfervations of Dr. Hull, upon a fingular cafe of difeafe in the bowels, which was publifhed by me in the fecond volume of the Medical and Chirurgical Tranfactions. I feel no difficulty in acknowledging that his explanation of the cafe is more fatisfactory than my own; and as it is fupported by an examination of the difeafed parts after death, in a cafe which bears a ftrong analogy to mine, I think it highly probable that his is the true explanation.

If, therefore, the volume in which my paper is publifhed fhall ever come to a fecond edition, I fhall take care to correct my miftake. I am, &c.

*Great Windmill Street, Jan.* 11, 1802. M. BAILLIE.

